# Medication prescribing errors in a pediatric inpatient tertiary care setting in Saudi Arabia

**DOI:** 10.1186/1756-0500-4-294

**Published:** 2011-08-14

**Authors:** Majed I Al-Jeraisy, Menyfah Q Alanazi, Mostafa A Abolfotouh

**Affiliations:** 1 King Abdullah International Medical Research Center, King Saud Bin-Abdulaziz University for Health Sciences, Riyadh, Saudi Arabia; 2 Drug Policy & Economic Center, King Abdulaziz Medical City, Riyadh, Saudi Arabia

**Keywords:** medication errors, prescriptions, pediatric, in-patient, Saudi Arabia

## Abstract

**Background:**

Medication errors (MEs) are among the most common types of medical errors and one of the most common and preventable causes of iatrogenic injuries. The aims of the present study were; (i) to determine the incidence and types of medication prescribing errors (MPEs), and (ii) to identify some potential risk factors in a pediatric inpatient tertiary care setting in Saudi Arabia.

**Findings:**

A five-week retrospective cohort study identified medication errors in the general pediatric ward and pediatric intensive care unit (PICU) at King Abdulaziz Medical City (KAMC) through the physical inspection of physician medication orders and reviews of patients' files. Out of the 2,380 orders examined, the overall error rate was 56 per 100 medication orders (95% CI: 54.2%, 57.8%). Dose errors were the most prevalent (22.1%). These were followed by route errors (12.0%), errors in clarity (11.4%) and frequency errors (5.4%). Other types of errors were incompatibility (1.9%), incorrect drug selection (1.7%) and duplicate therapy (1%). The majority of orders (81.8%) had one or more abbreviations. Error rates were highest in prescriptions for electrolytes (17.17%), antibiotics (13.72%) and bronchodilators (12.97%). Medication prescription errors occurred more frequently in males (64.5%), infants (44.5%) and for medications with an intravenous route of administration (50.2%). Approximately one third of the errors occurred in the PICU (33.9%).

**Conclusions:**

The incidence of MPEs was significantly high. Large-scale prospective studies are recommended to determine the extent and outcome of medication errors in pediatric hospitals in Saudi Arabia.

## Introduction

Medication errors (MEs) are one of the most common types of medical errors and one of the most common and preventable causes of iatrogenic injuries [1]. MEs contribute to the morbidity and mortality of hospitalized patients. In the USA, MEs have been found to be responsible for 7,000 patient injuries per year, with a similar incidence and consequences in the UK [2,3]. Approximately one third of adverse drug events (ADEs) are associated with medication errors and are thus preventable [4]. MEs occur in 6.5 of 100 adult hospital admissions and 5 of 100 adult medication orders [5].

When medication errors occur, pediatric patients have a much higher risk of death than adults [6]. There are many factors that put children at a greater risk for medication errors, such as their variations in age and weight, high intra-patient variability, rapid changes in the pharmacokinetic properties of drugs in children, and the frequent use of "off-label" indications in children [1,7-9]. Reports of MEs in children are becoming more common, yet the findings of many of the reports are conflicting [10]. In a systematic review, Miller et al. [11] found that 5 to 27% of inpatient orders contained errors.

Creating a prescription is an early step in medication use; therefore, reviewing orders and prescriptions by pharmacists and nurses is critical for detecting errors and preventing adverse impacts on patients [12]. In pediatrics, the prescribing and ordering phases, followed by the administration phase, are associated with the most errors (usually dosing errors) [1,3,11]. Prescription errors occur at a rate of 3 to 20% of all prescriptions in hospitalized pediatric patients and 10.1% of children seen in emergency departments [8,13]. In the Gulf region, few studies have explored the significance of medication errors in pediatric patients [14,15], and none of these studies has targeted pediatric inpatients. The electronic prescription system implemented by government health care facilities in Dubai has helped reduce medication errors by 50% [15]. The aims of the present study were to identify, in a pediatric inpatient tertiary care setting in Saudi Arabia, (i) the incidence and types of medication prescribing errors (MPEs) and (ii) some potential risk factors for MPEs.

## Materials and methods

### Study Setting

This study was conducted in the general pediatric wards and the pediatric intensive care unit (PICU) of King Abdulaziz Medical City (KAMC), which is a tertiary care hospital in Riyadh, the capital of Saudi Arabia. The pediatric/neonatal care units at KAMC are composed of general pediatric wards; medical, surgical and cardiac intensive care units; and oncology and pediatric emergency units. The total capacity of these wards is approximately 280 beds, which represents approximately 30% of the overall hospital capacity.

### Study design

This was a retrospective cohort study of physician medication orders over a period of five weeks in King Abdulaziz Medical City, Riyadh, Saudi Arabia.

### Study population

This study included all of the medication orders that were prescribed in the general pediatric wards -for children up to 14 years of age - and the pediatric intensive care unit (PICU) during the designated study period. The following orders were not included in the study: orders from the pediatric cardiac wards, the pediatric oncology wards, or the neonatal intensive care unit (NICU); orders corrected by the pharmacist prior to the review; errors in medication reorders; doses of antipyretics that were no more than 30% above the maximum dose; and verbal orders.

### Measurement

The medication orders were written by the pediatric medical staff of residents, fellows and consultants. They used either standard blank physician order sheets, a number of special order sheets (e.g., an antibiotics order sheet or an immunoglobulin order sheet), or pre-printed order-dependent protocols and clinical pathways. Copies of these original orders were sent to the pharmacy via fax or carried by the nurses or pharmacy aides. All of the medication orders were reviewed by staff pharmacists and entered into a computerized pharmacy data system prior to dispensation. The information on each medication order, including the date of birth, the diagnosis upon admission, any reported medication allergies, body weight, and a complete medication profile for the present hospitalization, was reviewed by the pharmacist. Two clinical pharmacists covered all of the orders for the pediatric patients.

Data were collected daily, and the following were examined: (1) the types of errors in terms of the drug selection, dose, frequency, route, order clarity, incompatibility, and therapy duplication; (2) the drug route of administration; (3) the hospital location; (4) the patient's age; and (5) the patient's gender. The use of unapproved abbreviations was recorded. All of the medication prescribing errors were then classified into two main categories: (i) potentially harmful errors (including all potential ADEs) and (ii) non-harmful errors (e.g., missing information, missing weight, illegibility, abbreviation errors, and trailing zeros) [8]. The pediatric staff was not informed of the study during the data collection period to assure the validity of the results.

### Operational definitions

A prescription error was defined as an incorrect or inappropriate drug selection (based on indications, contraindications and other factors), dose, route, rate of administration, or frequency. A prescription error also included illegible handwriting, an incomplete order (missing the dose, route, or frequency), incompatibility, incorrect instructions for using the drug product, and the use of non-standard nomenclature or abbreviations that requires further interpretation [4,11,12,16,17]. Because the definition of a medication error is non-uniform across different studies, the literature was thoroughly reviewed, and the categories were allocated accordingly.

### Ethical issues

This study was approved by the research committee at King Abdullah International Medical Research Center, King Saud Bin-Abdulaziz University for Health Sciences, Riyadh, Saudi Arabia.

## Results

### Types and incidence of medication errors

In the study period, a total of 2,380 medication orders were analyzed. In these, 1,333 medication errors were found, representing 56% (95% CI: 54.2, 57.8), as shown in table 1. The majority of these errors were classified as potentially harmful (1,051, 78.8%). Regarding the type of error, the incidence of dose errors was the highest (22.1%), followed by route errors (12.0%), order clarity (11.4%) and dose frequency (5.4%). Other types of errors were incompatibility (1.9%), incorrect drug selection (1.7%) and duplicate therapy (1%). Miscellaneous errors (0.4%) included drug-drug interactions, drug-food interactions, and an incorrect duration of therapy or monitoring.

**Table 1:** Frequency (%) and incidence of medication prescription errors according to type of error at King Abdulaziz Medical City, Riyadh, Saudi Arabia

Types of error	No. of errors	%	Incidence (95% CI)
**(A). Potentially harmful errors:**			22.1 (9.0, 25.2)
**Dose**			
Overdose	249	47.3	
Missed dose	186	35.4	
Underdose	79	15.0	
Undecided dose	12	2.3	
Subtotal *	526	**39.4**	

**Route**			12.0 (8.5, 15.5)
Missed route	271	94.8	
Undecided route	11	3.8	
Inappropriate route	4	1.4	
Subtotal *	286	**21.4**	

**Frequency**			5.4 (1.6, 9.2)
Missed frequency	95	73.6	
High frequency	17	13.2	
Low frequency	11	8.5	
Undecided frequency	6	4.7	
Subtotal *	129	**9.7**	

**Incompatibility**	45	**3.4**	1.9 (-2.1, 5.9)

**Wrong drug selection**	41	**3.1**	1.7 (-2.2, 5.6)

**Duplicate therapy**	24	**1.8**	1.00 (-3.1, 4.1)

**(B) Non-harmful errors:**			
**Clarity**	272	**20.4**	11.4 (7.8, 15.0)

**Miscellaneous**	10	**0.8**	0.4 (-5.8, 6.6)

**TOTAL**	1333	**100.0**	56.0 (54.2,57.8)

• Percentages were calculated from the total number of errors (N = 1,333).

Dose errors constituted more than one third of the errors (39.4%), with overdose as the main type of dose error (47.3%). A missed route was the main type of route error (94.8%), while a missed frequency was the main type of frequency error (73.6%). Medication prescription errors occurred the most frequently in males (64.5%), infants (44.5%) and intravenous routes of administration (50.2%). Approximately one third of the errors occurred in the PICU (33.9%) (table 2).

**Table 2:** Frequency (%) of medication prescription errors according to potential risk factors at King Abdulaziz Medical City, Riyadh, Saudi Arabia

Characteristics	No. of errors	%
**Gender**		
Male	860	64.5
Female	473	35.5
**Total**	1333	**100**

**Age group**		
Less than 1 year	593	44.5
1-3 years	249	18.7
4-6 years	167	12.5
7-9 years	43	3.2
10-12 years	281	21.1
**Total**	1333	**100**

**Administration route**		
Intravenous	669	50.2
Oral	342	25.7
Inhalation	222	16.6
Topical	60	4.5
Subcutaneous	24	1.8
Intramuscular	16	1.2
**Total**	1333	**100**

**Location**		
PICU	452	33.9
Pediatric wards	881	66.1
**Total**	**1333**	**100**

PICU: pediatric intensive care unit.

### Errors and medication category

**Figure 1:**
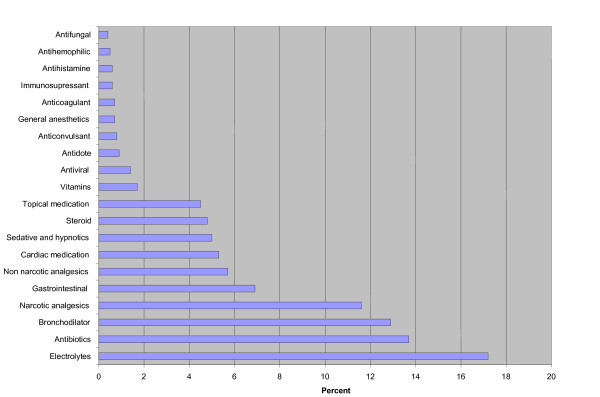
**Incidence (%) of medication errors in the different medication categories**.

Figure 1 shows that the largest number of errors were associated with prescribing electrolytes (17.2%), followed by antibiotics (13.7%), bronchodilators (12.9%), narcotic analgesics (11.6%), gastrointestinal medications (6.9%), non-narcotic analgesics (5.7%), cardiac medications (5.3%), sedatives and hypnotics (5%), steroids (4.8%), topical medications (4.5%) and vitamins (1.7%).

**Figure 2:**
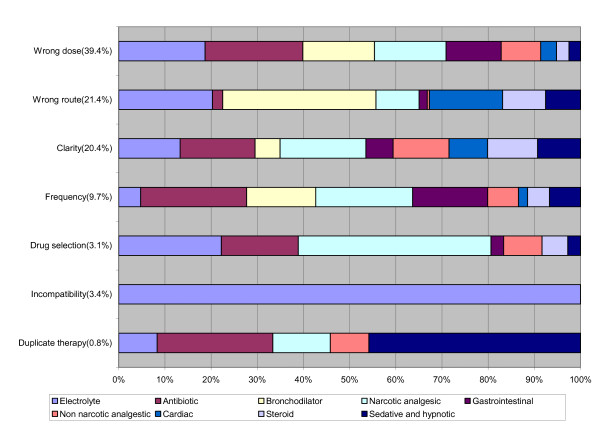
**Types of medication errors by medication category**.

Figure 2 shows that the majority of incompatibility errors (97%) occurred in prescribing electrolytes, while drug selection errors were committed mainly in prescribing narcotic analgesics (37%). Dose errors were committed in prescribing antibiotics (18%), electrolytes (16%), bronchodilators and narcotic analgesics (13% each). Route errors were represented in the prescription of bronchodilators (30%), electrolytes (18%), cardiac medications (14%) and steroids (8%). Frequency errors were committed in prescribing antibiotics (18%), narcotic analgesics (16%), gastrointestinal medications (12.6%), and bronchodilators (12%). Duplicate therapy errors were committed mainly in prescribing sedatives and hypnotics (42%) and antibiotics (23%).

Using the list of dangerous abbreviations of the Joint Commission, the study found that these abbreviations were highly prevalent (81.8%). They were in the form of QD (11.4%), a trailing zero after decimal point (e.g., 1.0 mg; 4.9%), a naked decimal point (e.g.,.5 mg; 2.6%), U (2.5%), and IU (1.9%). Incorrect drug abbreviations were observed in 17.1% of all of the prescriptions.

## Discussion

Medication errors, which are common when prescribing medication [18], are a serious and potentially harmful problem. Of the 2,380 medication orders that were analyzed in the present study, 1,333 errors (56%) were identified. The majority of these errors were classified as potentially harmful (1,051, 78.8%). This figure is much higher than the figures of 4.9 and 4.5 errors per 1,000 medication orders reported by Foli [19] for two large pediatric hospitals in the US and the figures of 5.9 per 1,000 reported by Lesar [20], 5.7% reported by Kaushal et al. [4], and 5.7% reported by Fortescue et al. [18]. Two more recent studies of medication errors in pediatric intensive care units by Potts [8] and Cimino [21] found that 3.4 and 11.1% of orders had at least one prescription error. However, the definition of medication error was non-uniform across these studies [10]. Both Potts [8] and Fortescue et al. [10] appeared to use a broader definition of medication error, as errors in both studies included all types of omissions, such as omissions in the patient's weight or prescriber's name [11]. These definitions differ from the definition of an omission in the present study, which only included a missing dose, route or frequency.

In general, dosing errors are the most common type of medication error in children, with overdoses generally outnumbering under-doses [4,10,20]. In this study, dosing errors were the most common type of error, followed by incorrect route, order clarity, and frequency.

Medication errors are more common in sicker patients with urgent and complex medical conditions [3,22]. In a previous study, although the total error rate per 100 orders was similar across different settings, serious errors were much more common in intensive care environments [23]. In the present study, approximately one third of the errors occurred in the PICU. This figure was higher than the figure reported by Cimino (11.1%) [21], and comparable to the figure (33.1%) reported by Potts [8]. When such a patient needs urgent care, there is often insufficient time to verify the correct dosage [24].

According to Foli [19], pediatric patients (2 years old or less) and pediatric ICU patients received the greatest proportion of errant orders. In the present study, 44.5% of all errors were in infants less than one year old. Children (< 5 years old) were at the highest risk of errors, and the risk for infants (< 1 year old) was more than double that of the next risk-prone group (aged 65 to 70)[19].

The use of abbreviations in prescribing medication has recently received much attention and has become an international concern as one of the major causes of medication errors [16,25,26]. In the present study, abbreviations were used in 82% of all orders. Abbreviations were in the form of QD (11.4%), a trailing zero after the decimal point (.9%), and a naked decimal point (2.6%). Incorrect abbreviations were found in 17% of all prescriptions. These figures were compared to those of Potts [8], who also found drug abbreviation errors (6.04%) and the use of a trailing zero after the decimal point (0.81%).

The administration route ranked second as a medication error in the present study, representing 21.4% of all errors. This finding was similar to that of a previous study [4] in which the administration route represented 18% of all errors. The intravenous route was most commonly associated with medication errors. This finding might explain the highest prevalence of errors in prescribing electrolytes and the finding that PICU errors were more common.

## Limitations

The present study has some limitations. First, the study was only conducted in one setting that does not represent other health care settings in Saudi Arabia. Thus, the findings cannot be generalized. Second, the study period was short (5 weeks), and most of the orders were collected during the day shift. It was difficult to collect the orders during the evening and night shifts, during which more serious mistakes may occur. Third, there was no follow-up studying the consequences of these errors (i.e., deaths and/or adverse events). Fourth, the incidence of errors was estimated based on the number of errors and not the number of orders with one or more errors, which might overestimate the incidence of errors in this study, making the comparison with other studies difficult.

Aside from these limitations, we can conclude the following:

1. The incidence of medication prescription errors in this tertiary care setting was significantly high. Dosing errors were the most common type of error, followed by the wrong route, order clarity, and the wrong frequency. Approximately one third of the errors occurred in the PICU, and one half occurred in infants less than one year old.

2. Pediatricians should help hospitals develop effective programs to safely provide medications, report medication errors, eliminate barriers to reporting medication errors, encourage a non-punitive reporting culture and create an environment of medication safety for all hospitalized pediatric patients.

3. The development, implementation, and assessment of a computerized physician order entry system, clinical decision-support systems, ward-based clinical pharmacists, and improved communication among physicians, nurses, and pharmacists are recommended.

4. Large-scale prospective studies are recommended to determine the extent and outcomes of medication errors in pediatric hospitals in Saudi Arabia.

## Competing interests

The authors declare that they have no competing interests.

## Authors' contributions

All of the authors contributed to the design and execution of the study and the analyses. MAA and MQA were actively involved in writing the manuscript. MIA commented on the drafts. All of the authors read and approved the final manuscript.
